# Supplementary material for: Mutant CEBPA promotes tolerance to inflammatory stress through deficient AP-1 activation
Source: Nat Commun. 2025 Apr 12;16:3492. doi: 10.1038/s41467-025-58712-7 (PMC11993602; doi:10.1038/s41467-025-58712-7)
Supplement: Supplementary file 1 — Supplementary Information [file 41467_2025_58712_MOESM1_ESM.pdf]

SUPPLEMENTARY INFORMATION

Mutant CEBPA promotes tolerance to inflammatory stress through deficient AP-1 activation

Maria Cadefau-Fabregat[1,2,3], Gerard Martínez-Cebrián[1,†], Lucía Lorenzi[1,†], Felix D. Weiss[4,‡], Anne-Katrine Frank[5,6,‡], José Manuel Castelló-García[7], Eric Julià-Vilella[1,3], Andrés Gámez-García[7], Laura Yera[1], Carini Picardi Morais de Castro[1,3], Yi-Fang Wang[8,9], Felix Meissner[4], Alejandro Vaquero[7], Matthias Merkenschlager[8,9], Bo T. Porse[5,6,10], Sergi Cuartero[1,2*]

[1] Josep Carreras Leukaemia Research Institute (IJC), Badalona, Spain

[2] Germans Trias i Pujol Research Institute (IGTP), Badalona, Spain

[3] Doctoral Program in Biomedicine, Universitat de Barcelona (UB), Spain

[4] Institute of Innate Immunity, Department for Systems Immunology and Proteomics, Medical Faculty, University Hospital Bonn, University of Bonn, 53127 Bonn, Germany

[5] The Finsen Laboratory, Copenhagen University Hospital - Rigshospitalet, Copenhagen, Denmark

[6] Biotech Research and Innovation Centre (BRIC), Faculty of Health Sciences, University of Copenhagen, Copenhagen, Denmark

[7] Chromatin Biology Laboratory, Josep Carreras Leukaemia Research Institute (IJC), Badalona, Spain

[8] MRC London Institute of Medical Sciences, Institute of Clinical Sciences, Faculty of Medicine, Imperial College London, Du Cane Road, London W12 0NN, UK

[9] Institute of Clinical Sciences, Faculty of Medicine, Imperial College London, Du Cane Road, London W12 0NN, UK

[10] Department of Clinical Medicine, University of Copenhagen, Copenhagen, Denmark

[†] These authors contributed equally

[‡] These authors contributed equally

*Correspondence to: scuartero@carrerasresearch.org

SUPPLEMENTARY FIGURES

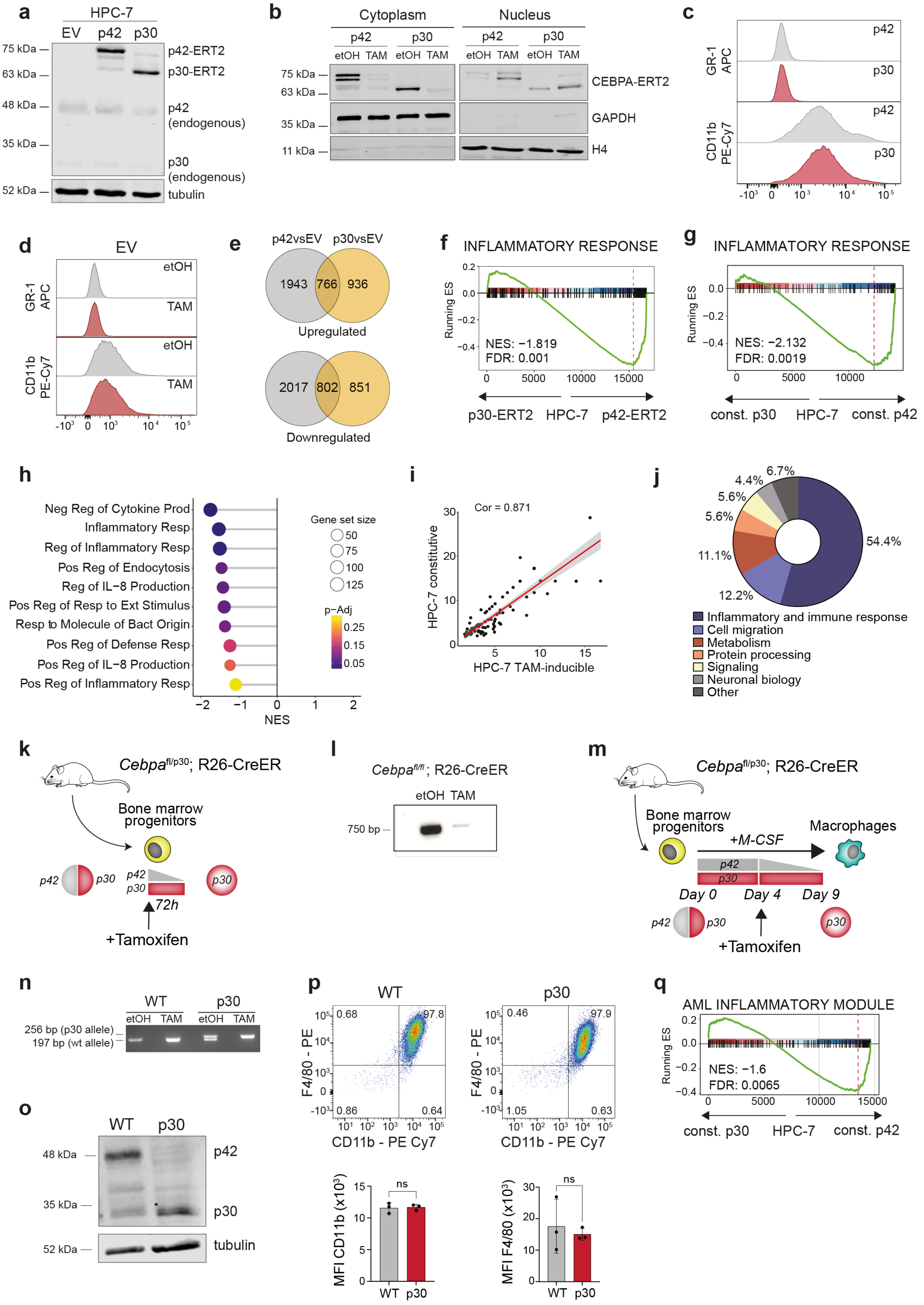

Supplementary Figure 1. Models of p30- and p42-expressing cells. a, Western blot showing CEBPA p30-ERT2 and p42-ERT2 overexpression in HPC-7 cells as well as endogenous CEBPA levels. **b**, Fractionation of HPC-7 p42-ERT2 and HPC-7 p30-ERT2 before and after nuclear translocation induced with tamoxifen (TAM), ethanol (etOH) was used as control. **c**, Flow cytometry histograms of GR-1 and CD11b of HPC-7 cells overexpressing p42 and p30 before treatment with tamoxifen. **d**, Flow cytometry histograms of GR-1 and CD11b of empty vector (EV) HPC-7 cells before and after treatment with tamoxifen (TAM), ethanol was used as control. **e**, Overlap between upregulated and downregulated genes in p42 and p30 HPC-7 compared to an empty vector (EV) control. p-value<0.05 and log2FC < |0|. **f**, GSEA of inflammatory response genes in p30 vs p42 HPC-7 cells. **g**, GSEA of inflammatory response genes in p30 vs p42 (constitutive expression) HPC-7. **h**, GSEA in HPC-7 expressing constitutive p30 vs p42 of GO terms enriched in downregulated genes in HPC-7 p30 (Figure 1b). **i**, Correlation plot of significantly downregulated GO terms (p30 vs p42) in HPC-7 expressing constitutive and tamoxifen-inducible *Cebpa*. Linear regression with standard error. **j**, Classification by function of enriched GO terms in common downregulated genes to TAM-inducible and constitutive p30 HPC-7. **k**, Schematic of the experimental model: bone marrow hematopoietic progenitors were isolated by lineage depletion of bone marrow cells from *Cebpa*$^{Fl/p30}$; R26-CreER mice. Deletion of the wild-type allele was achieved by culturing the cells in 400nM tamoxifen for 72h. **l**, PCR of *Cebpa* genetic deletion in *Cebpa*$^{Fl/Fl}$; R26-CreER bone marrow hematopoietic progenitors after 72h of tamoxifen (TAM) treatment. Cells were treated with ethanol (etOH) as control. **m**, Schematic of the experimental model: tamoxifen was added at day 4 of macrophage differentiation to induce genetic deletion of the wild-type allele while ensuring that both genotypes were terminally differentiated macrophages. **n**, Confirmation of the genetic deletion of the wild-type allele by PCR in *Cebpa*$^{Fl/p30}$; R26-CreER (p30) macrophages at day 9 of differentiation and treated with tamoxifen (TAM) at day 4. *Cebpa*$^{Fl/Fl}$ (WT) mice were used as control. Cells were treated with ethanol (etOH) as control. **o**, CEBPA western blot of *Cebpa*$^{Fl/Fl}$ (WT) and *Cebpa*$^{Fl/p30}$; R26-CreER (p30) macrophages at day 9 of differentiation. **p**, FACS plot of F4/80 and CD11b in *Cebpa*$^{Fl/Fl}$ (WT) and *Cebpa*$^{Fl/p30}$; R26-CreER (p30) macrophages at day 9 of differentiation. Quantification of the mean fluorescence intensity (MFI) of CD11b and F4/80 is shown in the bar plots at the bottom. Two-sided unpaired t-test, ns: P-value>0.05, mean +/- SEM. **q**, GSEA of the AML inflammatory module in p30 vs p42 (constitutive expression) HPC-7. **(k, m)** Mouse icon created in BioRender. C, S. (2025) https://BioRender.com/o35p709. All panels show data representative of n=3 biological replicates. Source data are provided as a Source Data file.

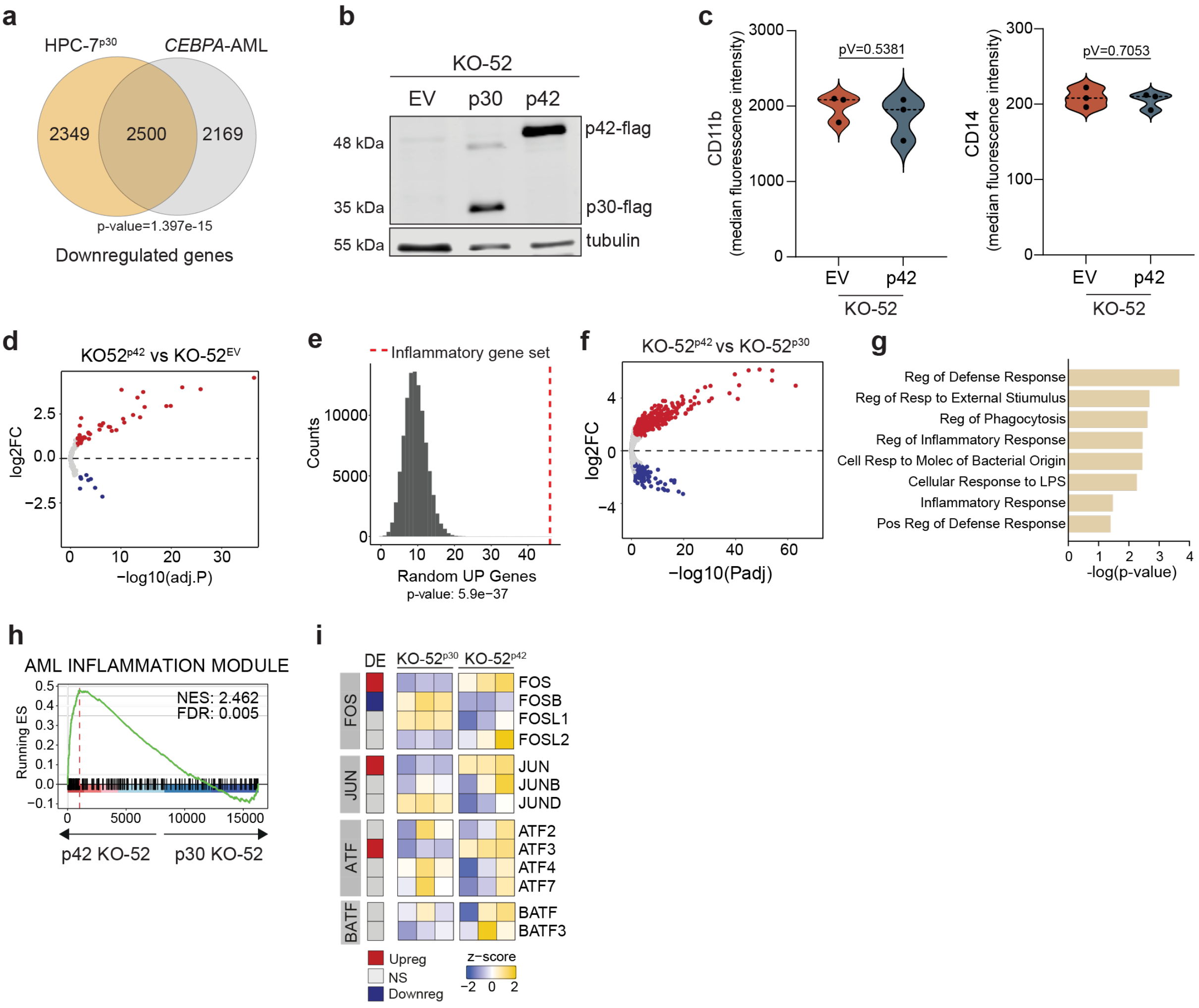

Supplementary Figure 2. CEBPA-mutated AML. a, Overlap between downregulated genes in p30 HPC-7 and in *CEBPA*$^{bi/NT}$-mutated AML (hypergeometric test). **b**, Overexpression of wild-type p30 and p42 in KO-52 cells compared to KO-52 cells transfected with an empty vector. **c**, Quantification of the median fluorescence intensity of CD11b (left) and CD14 (right), analyzed by FACS 48h after transfection of an empty vector or wild-type p42 in KO-52 cells. Two-sided unpaired t-test; ns: P-value>0.05, dashed line represents median, n=3. **d**, Volcano plot of inflammatory genes comparing KO-52 transfected with p42 vs empty vector (EV). **e,** Permutation test counting the number of up-regulated genes from random gene sets with the same size as the number of expressed inflammatory genes (n=100000). Red dashed line indicates the number of up-regulated genes in the inflammatory gene set (46 genes). **f**, Volcano plot of deregulated genes in KO-52^{p42} vs KO-52^{p30}. Red: significantly upregulated genes, blue: significantly downregulated genes (adj. p-value <0.05, log2FC > |1|, Wald test using Benjamini-Hochberg test for adjustment for multiple comparisons, n=3 biological replicates). **g**, Significance of enrichment of inflammatory gene ontology terms enriched in KO-52^{p42} vs KO-52^{EV} (top ten) when comparing KO-52^{p42} vs KO-52^{p30} cells. Fisher's exact test. **h**,

GSEA of an AML inflammation signature[56] in KO-52^{p42} vs KO-52^{p30}. NES, normalized enrichment score. **i**, Expression (z-score) of AP-1 family members grouped by subfamily in three replicates (each individual column is a replicate) of KO-52^{p30} and KO-52^{p42}. Left column (DE) indicates the differential expression status. Red: upregulated genes, blue: downregulated genes, grey: non-significantly changed genes (p-adj < 0.05 log2FC < |0|, Wald test using Benjamini-Hochberg test for adjustment for multiple comparisons, n=3 biological replicates). Source data are provided as a Source Data file.

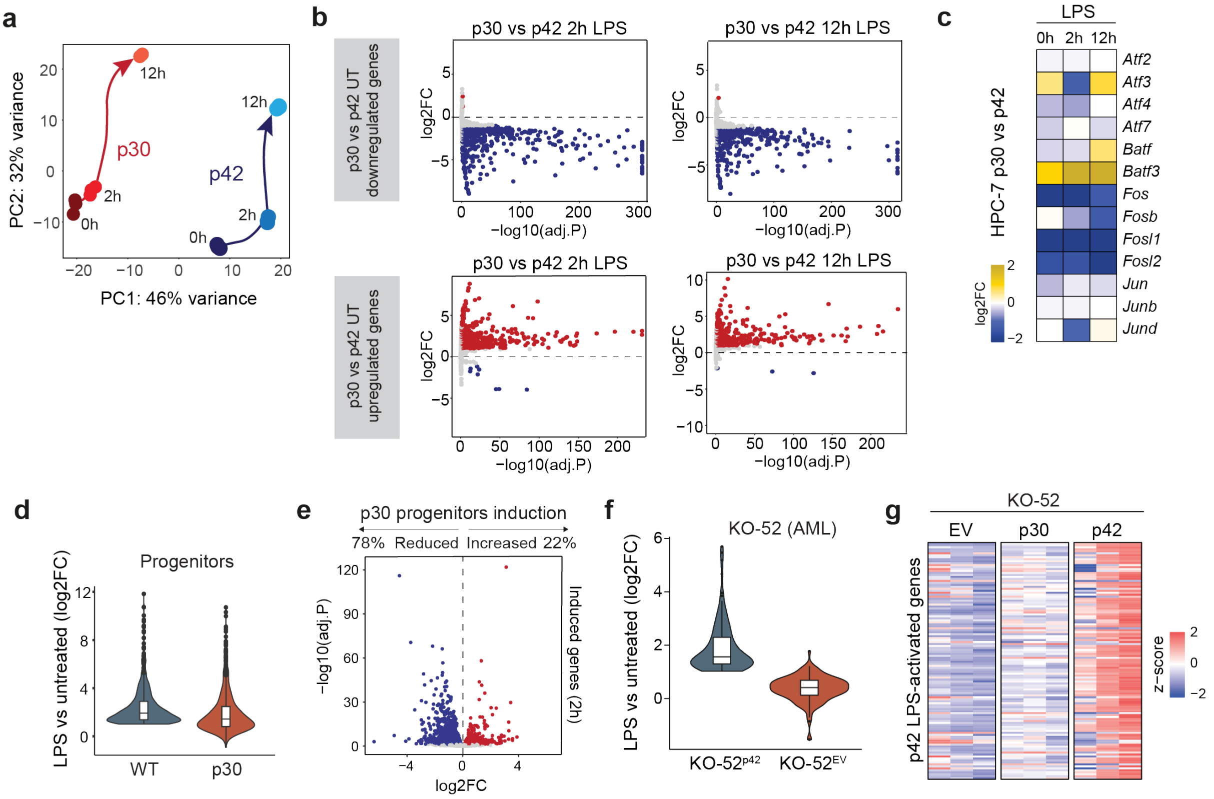

Supplementary Figure 3. Impaired LPS-activation dynamics in p30-expressing murine BM progenitors and in *CEBPA*-mutated AML a, PCA of RNA-seq samples from p30 and p42 HPC-7 cells over a time course of LPS stimulation. The three replicates are shown. **b**, Volcano plots of baseline upregulated and downregulated genes at 2h and 12h of LPS stimulation. adj.P <0.05, log2FC <|1|, Wald test using Benjamini-Hochberg test for adjustment for multiple comparisons. **c**, Heatmap of differential gene expression (log2FC) of AP-1 family genes comparing p42 vs p30 HPC-7 cells in an LPS time course **d**, Differential gene expression (log2 fold change) comparing WT LPS-activated genes at 2h of stimulation in bone marrow (BM) hematopoietic progenitors *Cebpa*$^{Fl/Fl}$ (WT) mice and *Cebpa*$^{Fl/p30}$; R26-CreER (p30) mice. Cells were treated with tamoxifen for 72h before LPS stimulation. **e**, Volcano plot of WT-induced genes at 2h LPS stimulation showing p30 vs WT BM hematopoietic progenitors. In red, upregulated genes and in blue, downregulated genes, adj.P <0.05, log2FC <|0|, Wald test using Benjamini-Hochberg test for adjustment for multiple comparisons. **f**, Differential gene expression (log2 fold change) comparing p42 LPS-activated cells versus untreated cells at 2h of stimulation in KO-52 cells transfected with p42 (KO-52^{p42}) and in KO-52 transfected with an empty vector control (KO-52^{EV}). **g**, Heatmap showing gene expression (z-score) of p42 LPS-activated genes (KO-52^{p42} 2h LPS vs untreated, adjP<0.05, log2FC >0) in KO-52 cells transfected with an empty vector (EV), p30 or p42 at 2h of LPS. All panels show data from n=3 biological replicates.

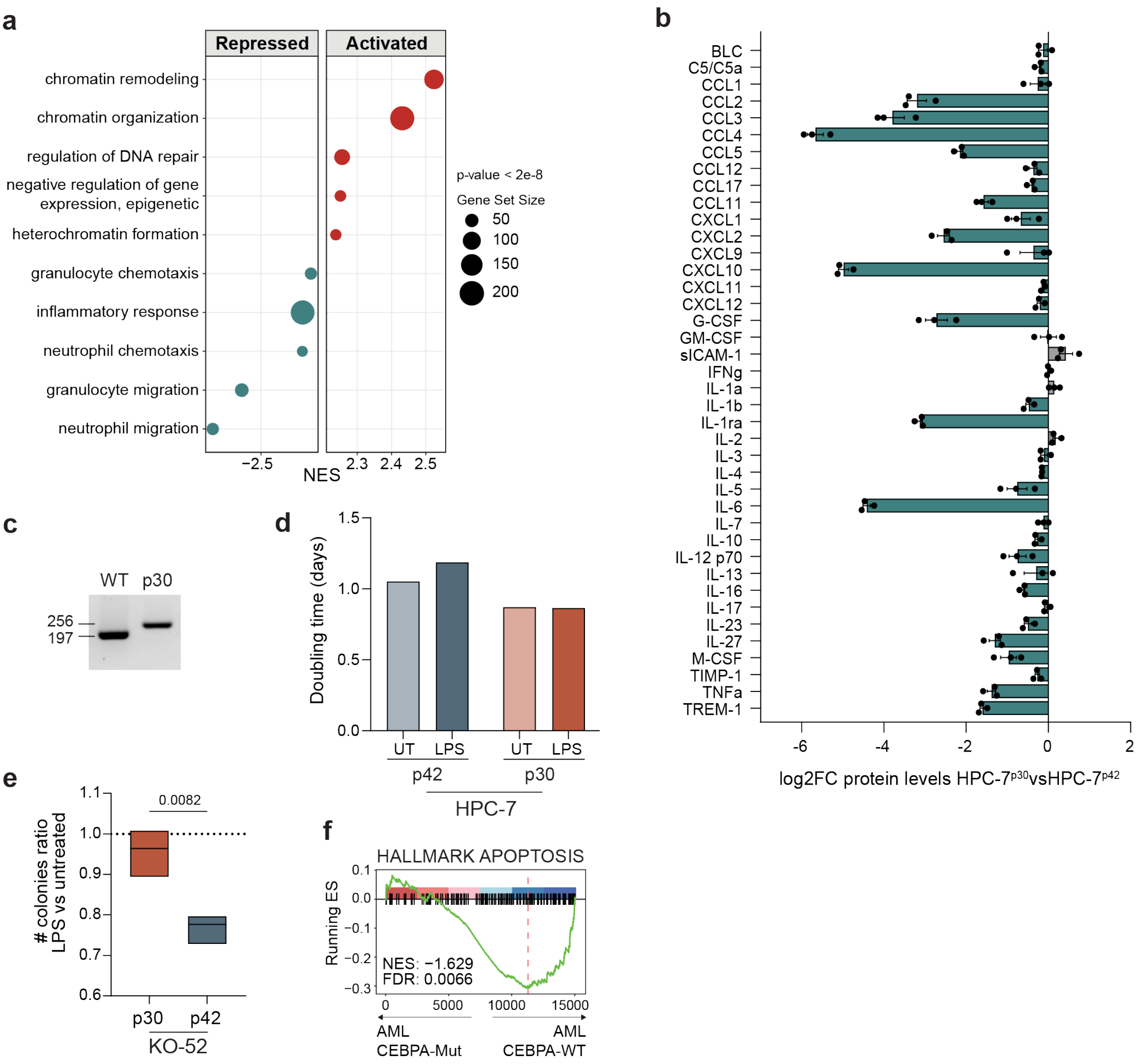

Supplementary Figure 4. Altered inflammatory phenotype of p30-expressing cells. a, Top 5 upregulated (red) and downregulated (blue) GSEA terms in p30 HPC-7secretome (detected by mass spectrometry) after 16h of stimulation with 100ng/mL LPS. NES, normalized enrichment score. **b**, Cytokine protein levels secreted in the media by p42 and p30 HPC-7 cells after 16h in LPS, detected by antibody-based cytokine array with a panel of 40 cytokines. Log2 fold change comparing expression in p30 against p42 is shown; in grey, non-significant changes (p-value>0.05) and in blue, downregulated in p30 HPC-7 (two-sided unpaired t-test, p-value <0.05, mean ± SEM, n=3). **c**, PCR showing *Cebpa* genetic deletion in methylcellulose-grown bone marrow progenitors from *Cebpa*$^{Fl/Fl}$ (WT) and *Cebpa*$^{Fl/p30}$; R26-CreER (p30) mice. **d**, Doubling time of p42 and p30 HPC-7 cells in the presence of LPS. **e**, Ratio of colony number in LPS vs untreated conditions in KO-52 p30 and KO-52 p42. **f**, GSEA

of hallmark apoptosis genes in *CEBPA*^{bi}- and *CEBPA*^{NT}-mutated AML compared to all other AML subtypes. Source data are provided as a Source Data file.

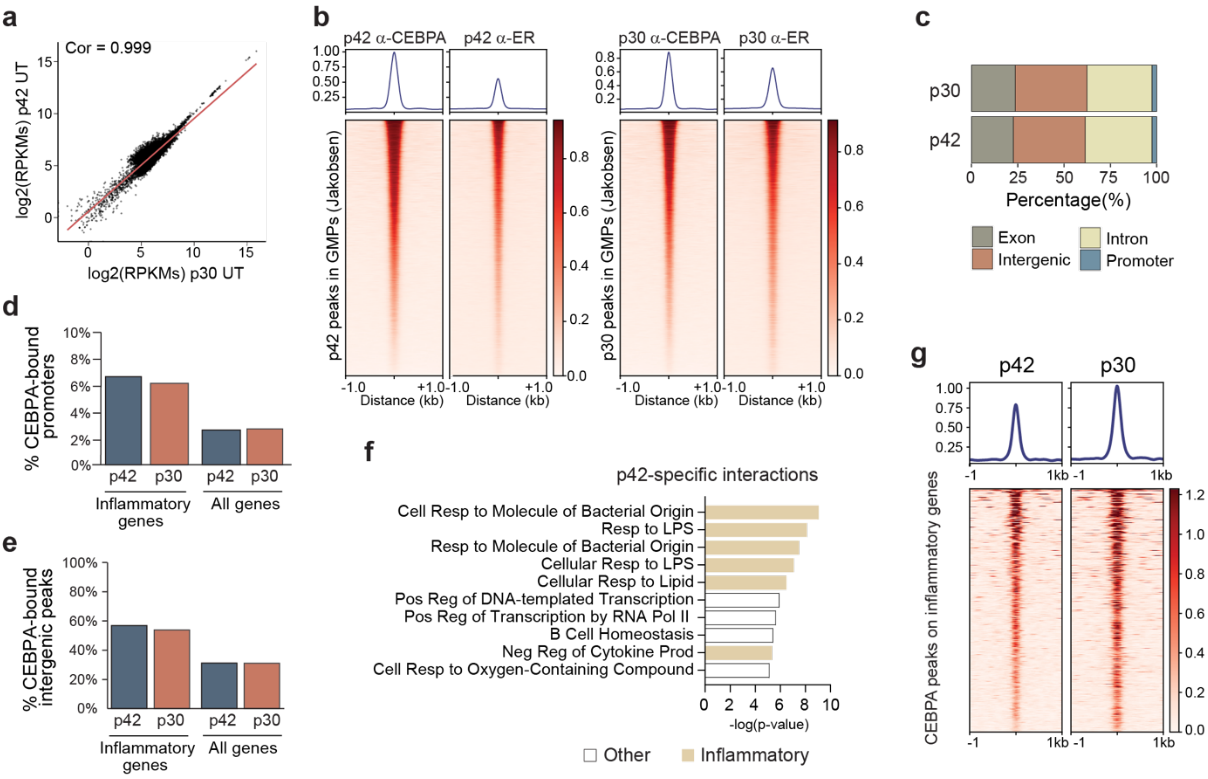

Supplementary Figure 5. CEBPA chromatin binding. a, Correlation of CEBPA ChIP-seq normalized signal in p42 and p30 HPC-7 cells. Linear regression with standard error. **b**, α-CEBPA and α-ER ChIP-seq signal enrichment at publicly available p42/p30 peaks from GMPs[35]. Left: HPC-7 p42-ERT2 cells. Right: HPC-7 p30-ERT2 cells. **c**, Genomic distribution of p30 and p42 ChIP-seq peaks from primary mouse GMPs[35]. **d**, Percentage of genes with CEBPA binding in their promoters in primary mouse GMP[35]. **e**, Percentage of genes most proximal to enhancers with CEBPA binding in primary mouse GMPs[35]; enhancers were defined as H3K27ac peaks that are not in promoters. **f**, Top 10 gene ontology terms enriched in genes from interactions stronger in p42 HPC-7 (p42-specific interactions) determined by H3K27ac HiChIP (n=2). Cells were treated with 100ng/mL LPS for 2h. Statistical significance assessed by Fisher's exact test. Resp: response, Pos: positive, Reg: regulation, Neg: negative, Prod: production. **g**, CEBPA ChIP-seq signal at inflammatory genes bound by both isoforms in HPC-7 cells.

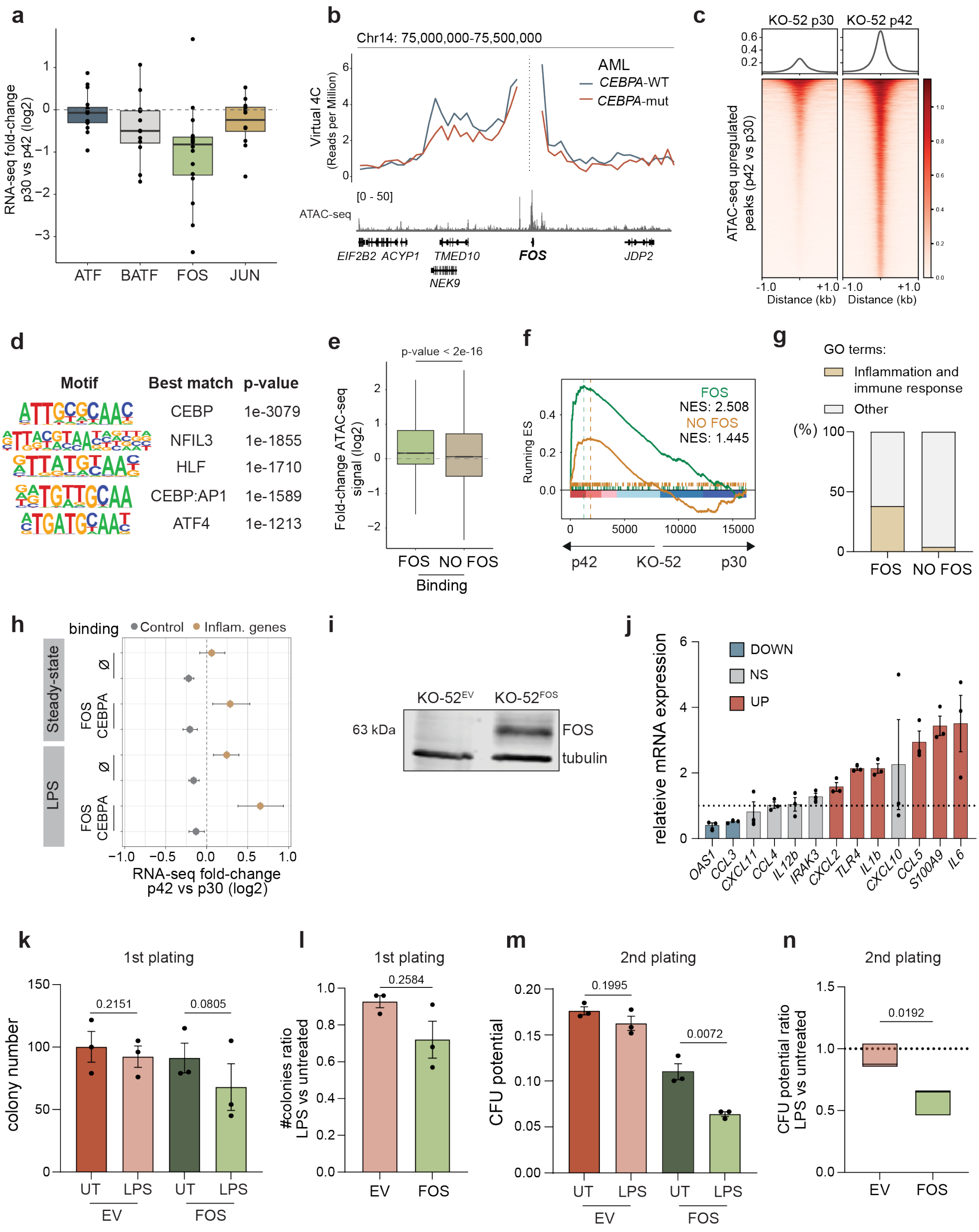

Supplementary Figure 6. FOS downregulation in CEBPA-mutant AML and rescue. a, Boxplot of the mean log2 fold change expression of the main AP-1 subfamilies members (*ATF*, *BATF*, *FOS* and *JUN*) in various cellular models: *CEBPA*$^{NT/bi}$-mutated AML compared to all other AML subtypes, p30 HPC-7 vs p42 HPC-7 and *Cebpa*$^{Fl/p30}$;R26-CreER (p30) vs *Cebpa*$^{Fl/Fl}$

(WT) macrophages and progenitors. **b**, Virtual 4C of the *FOS* gene locus in *CEBPA*-mutant and *CEBPA*-WT AML patient cells. Track underneath shows ATAC-seq signal. **c**, ATAC-seq signal of upregulated peaks (adj.P < 0.05, log2FC > 0) in p42 compared to p30 KO-52 cells shown in KO-52 transduced with p30 or p42. **d**, Top 5 motifs enriched (cumulative binomial distribution) in upregulated ATAC-seq peaks (adj.P < 0.05, log2FC > 0) in p42 vs p30 KO-52 cells. **e**, log2 fold change in accessibility (p42 vs p30 KO-52), peaks classified by sites bound by FOS or without FOS binding. Two-sided unpaired t-test, n=2 samples for ATAC-seq. **f**, GSEA of genes that had increased accessibility (adj.P <0.05, log2FC >1) at their promoters, separating them by the presence of FOS binding, in p42 vs p30 KO-52. NES, normalized enrichment score, p-value < 0.05. **g**, Classification of the top 50 enriched GO terms between inflammation and immune response- related terms and all the other categories in genes that had increased accessibility (adj.P <0.05, log2FC >1) at their promoters, separated by the presence of FOS. **h**, Expression by log2 fold change (p42 vs p30 KO-52) of hallmark inflammatory genes and housekeeping genes (control) grouped by co-binding of FOS and CEBPA (or no binding) at their promoters. Cells were treated with LPS for 2h or left untreated (steady-state). Error bars show mean ± CI. **i**, FOS overexpression in KO-52 cells by western blot, representative of three replicates. **j**, Quantitative RT-PCR of representative inflammatory genes in KO-52 cells transduced with FOS and treated with 100ng/mL LPS for 2h, expression relative to an empty vector control (dotted line) and normalized to *HPRT* and *RPL38*. In blue, downregulated genes; in red, upregulated (p-value<0.05) and in grey, non-significant changes (t-test, p-value>0.05), mean ± SEM, n=3. **k**, Number of colonies at day 14 from KO-52 cells with an empty vector (EV) or overexpressing FOS. Methylcellulose was supplemented with 1µg/mL LPS where indicated. **l**, Ratio of colony number in LPS vs untreated conditions in KO-52 EV and KO-52 FOS at day 14 of the first plating. **m**, Cumulative CFU potential for second plating of KO-52 EV and KO-52 FOS cells, methylcellulose was supplemented with 1µg/mL LPS where indicated. **n**, Ratio of the cumulative CFU potential in LPS vs untreated conditions in KO-52 EV and KO-52 FOS at day 14 of the second plating. **(k-n)** Significance assessed by two-sided unpaired t-tests, mean ± SEM, n=3. Source data are provided as a Source Data file.

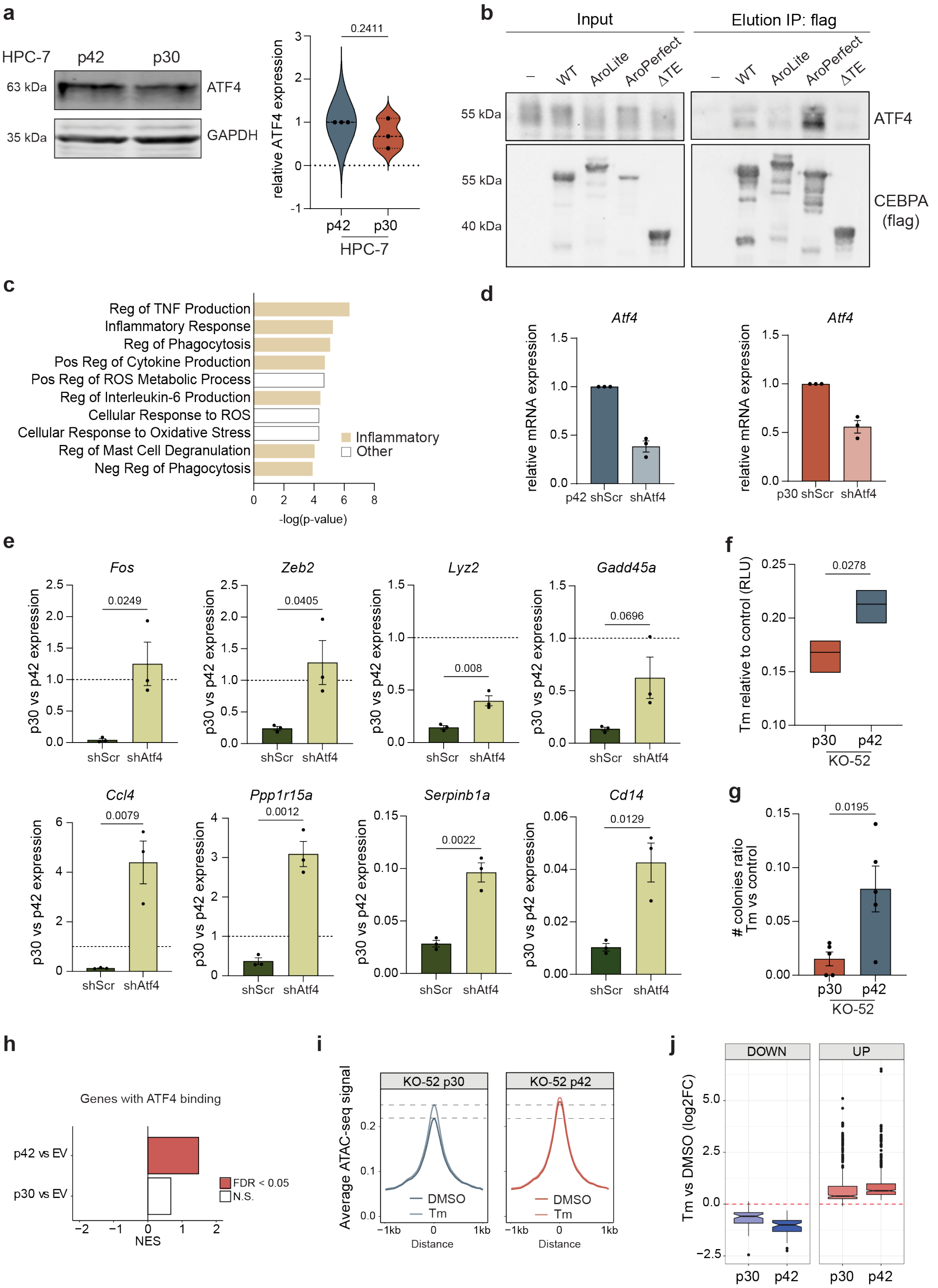

Supplementary Figure 7. Regulation of ATF4 by CEBPA isoforms. a, Western blot of ATF4 in HPC-7 cells expressing either p42 or p30 after treatment with tunicamycin (Tm) for

8h. Right panel: Expression is quantified by normalization to GAPDH and shown relative to levels in p42 cells. **b**, Immuno-blot against CEBPA-Flag and ATF4 of the immunoprecipitation of CEBPA-Flag in HEK293T cells overexpressing Flag-tagged WT CEBPA, CEBPA-AroLITE, CEBPA-AroPERFECT or CEBPA-ΔTEIII. **c**, Top 10 gene ontology terms enriched in genes co-regulated by ATF4 and p42. Reg: regulation, Pos: positive, ROS: reactive oxygen species, Neg: negative. Fisher's exact test. **d**, *Atf4* gene expression in HPC-7 p42 and HPC-7 p30 transduced with shScr or shRNA against *Atf4*, determined by RT-qPCR. **e**, Differential gene expression between HPC-7 p30 and p42 with shScramble (Scr) or an *Atf4* knockdown (shAtf4) of a panel of key dysregulated genes, quantified by RT-qPCR, data shown as mean +/- SEM. **f**, Cell viability measured with Cell Titer-Glo assay showing luminescence of KO-52 p30 and KO-52 p42 cells treated with 2μg/mL tunicamycin (Tm) for 5 days compared to DMSO treatment. **g**, Ratio of colony number in tunicamycin (Tm) vs untreated conditions in KO-52 p30 and KO-52 p42 at day 14. Tm was added to methylcellulose at 2μg/mL. Data shown as mean +/- SEM. **h**, GSEA of genes bound by ATF4 at their promoters and gene body, shown in KO-52 cells transfected with p42 vs EV and p30 vs EV. **i**, Average ATAC-seq signal at ATF4 binding sites in KO-52 cells transduced with p30 or p42 and treated with tunicamycin (Tm) for 8h or DMSO as control. Dotted lines indicate the top signal in p30 cells. **j**, log2 fold change (Tm vs DMSO) of Tm-responsive peaks in KO-52 transduced with p30 or p42. Tm-responsive peaks were defined as differentially accessible peaks after tunicamycin Tm in p42 KO-52 cells. DMSO was used as control of treatment. **(a, e-g)** Statistical significance by two-sided unpaired t-test, n=3 independent experiments. Source data are provided as a Source Data file.

SUPPLEMENTARY TABLES

Supplementary Table 1. *Overlap between HPC-7 and mouse primary progenitors*

		HPC-7 p30 vs p42			
		UP	DOWN	Unchanged	Total
Progenitors P30 vs WT	UP	142	5	242	389
	DOWN	11	202	137	350
	Unchanged	435	520	12459	13414

UP: adj.P < 0.05, log2FC >1. DOWN: adjP < 0.05, log2FC < -1. Genes that did not fit into these criteria are shown as Unchanged.

Supplementary Table 2. *Primers for RT-qPCR*.

**Gene**	**FORWARD sequence (5'-3')**	**REVERSE sequence (5'-3')**
HPRT	GACCAGTCAACAGGGGACAT	CTGCATTGTTTTGCCAGTGT
RPL38	TGGGTGAGAAAGGTCCTGGTC	CGTCGGGCTGTGAGCAGGAA
CCL3	CCTGCTCAGAATCATGCAGGT	CGTGTCAGCAGCAAGTGAT
CCL4	CTCCCAGCCAGCTGTGGTATTC	CAGGATTCACTGGGATCAGCAC
CCL5	GCTGCTTTGCCTACATTGCC	TTGACCTGTGGACGACTGC
CXCL2	CTTGTCTCAACCCCGCAT	CAGTTGGATTTGCCATTTTTCAG
CXCL10	TGATGCAGGTACAGCGTACAG	AGTGGCATTCAAGGAGTACC
CXCL11	ATGCAAAGACAGCGTCCTCT	ACAGTTGTTCAAGGCTTCCC
IL1b	ACCAAACCTCTTCGAGGCAC	TGGCTGCTTCAGACACTTGAG
IL12b	TGGATGCCGTTCACAAGCTC	AGCTGCAAGTTCTTGGGTGG
IL6	AGTCCTGATCCAGTTCCTGC	CTGGCATTTGTGGTTGGGTC
IRAK3	GAATTACTTTGGTCCTGGGCAC	TCTGAAGGACTCAACACTGCT
OAS1	GCGGACCCTACAGGAAACTT	TGTGCTGGGTCAGCAGAATCC
S100A9	TCTGCATTTGTGTCCAGGTC	GAATTCAAAGAGCTGGTGCG
Rpl32	ACCCCAGAGGCATTGACAAC	ATTGTGGACCAGGAACTTGC
Atf4	CCGGAAATTCGTCAACGAGC	AGATCGTCCTAAAGGCCCCA
Ccl4	AACCTAACCCCGAGCAACAC	AGGGTCAGAGCCCATTGGTG
Cd14	CTACCGACCATGGAGCGTG	TGAAAGCGCTGGACCAATCT
Cxcr4	CTCTGAGGCGTTTGGTGCTC	GAAGCAGGGTTCCTTGTTGG
Fos	ACGGAGAATCCGAAGGGAAC	TCAAGTTGATCTGTCTCCGCT
Gadd34	TCCTCTAAAAGCTCGGAAGGTACA	ATCTCGTGCAAACTGCTCCC
Gadd45a	CTGTGTGCTGGTGACGAACC	TCCATTCGGATGCCATCACC
Lyz2	ATGAACGTTGTGAGTTTGCCAG	ATTGCTCTCGTGCTGAGCTA
Serpinb1a	TCCTAGCTGTAAGTGGAGCC	TCCATGGTGAAGTCTCTTGCT
Zeb2	TACCTTCAGCGAAGCGACAC	GTTCCAGGTGGCAGGTCATT
