# Supplementary material for: Mutant CEBPA promotes tolerance to inflammatory stress through deficient AP-1 activation
Source: Nat Commun. 2025 Apr 12;16:3492. doi: 10.1038/s41467-025-58712-7 (PMC11993602; doi:10.1038/s41467-025-58712-7)
Supplement: Supplementary file 2 — Reporting Summary [file 41467_2025_58712_MOESM2_ESM.pdf]

nature portfolio

Corresponding author(s): Sergi Cuartero
Last updated by author(s): Mar 20, 2025

Reporting Summary

Nature Portfolio wishes to improve the reproducibility of the work that we publish. This form provides structure for consistency and transparency in reporting. For further information on Nature Portfolio policies, see our Editorial Policies and the Editorial Policy Checklist.

Statistics

For all statistical analyses, confirm that the following items are present in the figure legend, table legend, main text, or Methods section.

n/a	Confirmed	
☐	☒	The exact sample size (*n*) for each experimental group/condition, given as a discrete number and unit of measurement
☐	☒	A statement on whether measurements were taken from distinct samples or whether the same sample was measured repeatedly
☐	☒	The statistical test(s) used AND whether they are one- or two-sided<br>*Only common tests should be described solely by name; describe more complex techniques in the Methods section.*
☒	☐	A description of all covariates tested
☐	☒	A description of any assumptions or corrections, such as tests of normality and adjustment for multiple comparisons
☐	☒	A full description of the statistical parameters including central tendency (e.g. means) or other basic estimates (e.g. regression coefficient) AND variation (e.g. standard deviation) or associated estimates of uncertainty (e.g. confidence intervals)
☐	☒	For null hypothesis testing, the test statistic (e.g. *F*, *t*, *r*) with confidence intervals, effect sizes, degrees of freedom and *P* value noted<br>*Give P values as exact values whenever suitable.*
☒	☐	For Bayesian analysis, information on the choice of priors and Markov chain Monte Carlo settings
☒	☐	For hierarchical and complex designs, identification of the appropriate level for tests and full reporting of outcomes
☒	☐	Estimates of effect sizes (e.g. Cohen's *d*, Pearson's *r*), indicating how they were calculated

Our web collection on statistics for biologists contains articles on many of the points above.

Software and code

Policy information about availability of computer code

Data collection	For data collection, we used the following software:<br>BD FACSDiva v6.1.3<br>FlowJo v10.6.2<br>QuantStudio 7 v1.6.1
Data analysis	For data analysis, we used the following software and packages:<br>GraphPad Prism v10.4.1<br>FlowJo v10.6.2<br>ImageStudioLite v5.2.5 (Li-COR)<br>Microsoft Excel v16.95<br>bowtie2 v2.4.4<br>Subread v2.0.3<br>R v4.3<br>TrimGalore v0.6.6<br>MACS2 v2.2.5<br>bedtools v.2.31.1<br>Deeptools bamCoverage v.3.3.1<br>BWA-MEM software (v. 0.7.17)<br>FitHiChIP v10.0

ProteoWizard v. 3.0.21321
DIA-NN v1.8
limma v3.54.2
igv (integrated genome browser) v2.19.2
sva v3.46.0
HiCcompare v1.20.0
ClusterProfiler v4.6.2
csaw v1.32.0
GenomicInteractions v1.32.0
juicer tools v1.22.01
DESeq2 v1.38.3
Enrichr
Picard markDuplicates v2.26.11
Homer v4.11.1

For manuscripts utilizing custom algorithms or software that are central to the research but not yet described in published literature, software must be made available to editors and reviewers. We strongly encourage code deposition in a community repository (e.g. GitHub). See the Nature Portfolio guidelines for submitting code & software for further information.

Data

Policy information about availability of data

All manuscripts must include a data availability statement. This statement should provide the following information, where applicable:

- Accession codes, unique identifiers, or web links for publicly available datasets
- A description of any restrictions on data availability
- For clinical datasets or third party data, please ensure that the statement adheres to our policy

The data generated for this study has been deposited at GEO under accessions GSE270547 (ChIP-seq), GSE270549 (HiChIP), GSE288477 (ATAC-seq) and GSE270550 (RNA-seq). The mass spectrometry proteomics data have been deposited to the ProteomeXchange Consortium via the PRIDE partner repository with the dataset identifier PXD053800. All code used in this study is available from the authors.
The following previously publicly available data were used: ATF4 ChIP-seq peaks were computed from the dataset GSM881130, CEBPA and FOS peaks in KO-52 were computed from GSE211095, CEBPA ChIP-seq in GMPs from GSE118963. HiC from AML patients from GSE152136.
Source data are provided with this paper.

Research involving human participants, their data, or biological material

Policy information about studies with human participants or human data. See also policy information about sex, gender (identity/presentation), and sexual orientation and race, ethnicity and racism.

Reporting on sex and gender	Sex was not considered in the study of public human AML RNA-seq data, as the gene of interest (CEBPA) is autosomal and no reports indicate that CEBPA function is gender-biased.
Reporting on race, ethnicity, or other socially relevant groupings	Race was not considered in the study of public human AML RNA-seq data, as no reports indicate that CEBPA function is different in different populations.
Population characteristics	Human data from AML patients used are from the BEAT study and TCGA database. Mutational status was the only factor considered. Details on patient characteristics can be found in the databases [https://portal.gdc.cancer.gov] and [http://www.vizome.org/aml2/]
Recruitment	The data we used is public (BEAT and TCGA databases) and we did not participate in the recruitment and data collection.
Ethics oversight	The data we used is public (BEAT and TCGA databases) and we did not participate in the recruitment and data collection.

Note that full information on the approval of the study protocol must also be provided in the manuscript.

Field-specific reporting

Please select the one below that is the best fit for your research. If you are not sure, read the appropriate sections before making your selection.

[x] Life sciences [ ] Behavioural & social sciences [ ] Ecological, evolutionary & environmental sciences

For a reference copy of the document with all sections, see nature.com/documents/nr-reporting-summary-flat.pdf

Life sciences study design

All studies must disclose on these points even when the disclosure is negative.

Sample size	We used 3 biological replicates to allow statistical inference as is the consensus in the field, with the following exceptions: 2 replicates for HiChIP, 1 replicate for CEBPA and ATF4 ChIP-seq, 2 replicates for ATAC-seq, 2 replicates for co-IPs.

Data exclusions	No data were excluded from the analysis.
Replication	3 biological replicates (i.e. number of mice, number of individual experiments for a cell line) per group/condition were used to allow statistical inference for all experiments, with the following exceptions: 2 replicates for HiChIP, 1 replicate for CEBPA and ATF4 ChIP-seq, 2 replicates for ATAC-seq, 2 replicates for co-IPs. Measurements were taken from distinct samples, not the same sample measured repeatedly. Replication attempts were successful and the results are represented as mean +- SEM where indicated.
Randomization	No randomization method was used.
Blinding	No blinding was used. Blinding was not possible since the experimental procedures were performed by a single investigator. All samples were processed and analysed in the same way.

Reporting for specific materials, systems and methods

We require information from authors about some types of materials, experimental systems and methods used in many studies. Here, indicate whether each material, system or method listed is relevant to your study. If you are not sure if a list item applies to your research, read the appropriate section before selecting a response.

Materials & experimental systems

n/a	Involved in the study	
☐	☒	Antibodies
☐	☒	Eukaryotic cell lines
☒	☐	Palaeontology and archaeology
☐	☒	Animals and other organisms
☒	☐	Clinical data
☒	☐	Dual use research of concern
☒	☐	Plants

Methods

n/a	Involved in the study	
☐	☒	ChIP-seq
☐	☒	Flow cytometry
☒	☐	MRI-based neuroimaging

Antibodies

Antibodies used	CD11b-PE/Cy7, M1/70 (BioLegend 101215), 1:400 dilution<br>F4/80 - PE, T45-2342, (BD Pharmingen 565410), 1:400 dilution<br>Gr1 (Ly6G/C)-biotin, RB6-8C5, (BioLegend 108403), 1:400 dilution for flow cytometry, 1:100 for lineage depletion<br>human CD14-APC, TUK4, (130-091-243, Miltenyi biotec), 1:400 dilution<br>PE-Streptavidin (BioLegend 405203), 1:400 dilution<br>CEBPA D56F10 (8178, Cell Signaling), 1:1000 dilution for western blot, 0.5ug for ChIP-seq<br>FOS T.142.5 (MA5-15055, Thermo Fisher), 1:500 dilution<br>ATF4 B3 (118155, Cell Signaling), 1:1000 dilution for western blot, 0.7ug for ChIP seq<br>FLAG M2 (F1804, Sigma), 1:2000 dilution<br>α-tubulin B-5-1-2 (T6074, Sigma), 1:4000 dilution<br>GAPDH 6C5 (sc-32233, Santa Cruz Biotechnology), 1:1000 dilution<br>H3K27ac antibody (Abcam ab4729), 4ug for ChIP-seq<br>H3K4me1 (Diagenode, C15410194) 3ug for ChIP-seq<br>ER F-10 (sc-8002X and sc-8002, Santa Cruz Biotechnology), 1:500 dilution for western blot, 8ug for ChIP<br>H3K27ac antibody D5E4 (Cell Signaling, 8173), 300ng for HiChIP<br>H4 (ab10158, abcam), 1:1000 dilution<br>Biotin-Nk-1.1, PK136, (108703, BioLegend), 1:100<br>Biotin-CD4 RM4-5, (100507, BioLegend), 1:100<br>Biotin-CD3 145-2C11, (100303, BioLegend), 1:100<br>Biotin-CD8 52-6.7, (100703, BioLegend), 1:100<br>Biotin-CD11b M1/70, (101203,BioLegend), 1:100<br>Biotin-CD19 6D5, (115503,BioLegend), 1:100<br>Biotin-Ter119 TER-119 (clone), (116203, BioLegend), 1:100<br>Biotin-B220 RA3-6B2, (103203, BioLegend), 1:100
Validation	Antibodies used were validated by the commercial supplier for the application used and in the species analysed.<br>Cell signaling validations: https://www.cellsignal.com/about-us/cst-antibody-validation-principles?srsltid=AfmBOopazzE3FDZPTWUgGlZd825mHKH7SRqFK7YJYDIDCOIT6SgbcmEs<br>Sigma validation: https://www.sigmaaldrich.com/ES/en/technical-documents/technical-article/protein-biology/elisa/antibody-standard-validation<br>Abcam validation: https://www.abcam.com/en-us/stories/articles/how-we-validate-our-recombinant-antibodies?srsltid=AfmBOoqyVYjzv_ZnV14wUk0R9D5-q4hPvviQPlleTCznStf7lNVVzYLM<br>Santa Cruz Biotechnology validates by western blot, immunofluorescence and ChIP

Eukaryotic cell lines

Policy information about cell lines and Sex and Gender in Research

Cell line source(s)	HPC-7 cells: Kindly provided by Luciano di Croce's laboratory (CRG). Cell gender: female.<br>KO-52 cells: Purchased at the JCRB cell bank (JCRB0123). Cell gender: male.
Authentication	The lines were not authenticated.
Mycoplasma contamination	All cell lines tested negative for mycoplasma contamination (monthly testing).
Commonly misidentified lines (See ICLAC register)	None

Animals and other research organisms

Policy information about studies involving animals; ARRIVE guidelines recommended for reporting animal research, and Sex and Gender in Research

Laboratory animals	Mus musculus, C57BL/6 background, 13-16 weeks old.
Wild animals	The study did not involve wild animals.
Reporting on sex	Sex was not considered in the study design, as the gene of interest (CEBPA) is autosomal and no reports indicate that CEBPA function is gender-biased.
Field-collected samples	The study did not involve samples collected from the field.
Ethics oversight	Danish Animal Ethical Committee, the Danish Animal Experiments Inspectorate and the Department of Experimental Medicine at hte University of Copenhagen.

Note that full information on the approval of the study protocol must also be provided in the manuscript.

Plants

Seed stocks	*Report on the source of all seed stocks or other plant material used. If applicable, state the seed stock centre and catalogue number. If plant specimens were collected from the field, describe the collection location, date and sampling procedures.*
Novel plant genotypes	*Describe the methods by which all novel plant genotypes were produced. This includes those generated by transgenic approaches, gene editing, chemical/radiation-based mutagenesis and hybridization. For transgenic lines, describe the transformation method, the number of independent lines analyzed and the generation upon which experiments were performed. For gene-edited lines, describe the editor used, the endogenous sequence targeted for editing, the targeting guide RNA sequence (if applicable) and how the editor was applied.*
Authentication	*Describe any authentication procedures for each seed stock used or novel genotype generated. Describe any experiments used to assess the effect of a mutation and, where applicable, how potential secondary effects (e.g. second site T-DNA insertions, mosiacism, off-target gene editing) were examined.*

ChIP-seq

Data deposition

- [x] Confirm that both raw and final processed data have been deposited in a public database such as GEO.
- [x] Confirm that you have deposited or provided access to graph files (e.g. BED files) for the called peaks.

Data access links<br>*May remain private before publication.*	ChIP-seq<br>GEO accession GSE270547:<br>Go to https://www.ncbi.nlm.nih.gov/geo/query/acc.cgi?acc=GSE270547
Files in database submission	HPC-7, p30, LPS 2h, H3K27Ac, ChIP, R1<br>HPC-7, p30, LPS 2h, H3K27Ac, ChIP, R2<br>HPC-7, p30, LPS 2h, H3K27Ac, ChIP, R3<br>HPC-7, p30, UT, H3K27Ac, ChIP, R1<br>HPC-7, p30, UT, H3K27Ac, ChIP, R2<br>HPC-7, p30, UT, H3K27Ac, ChIP, R3<br>HPC-7, p42, LPS 2h, H3K27Ac, ChIP, R1<br>HPC-7, p42, LPS 2h, H3K27Ac, ChIP, R2

HPC-7, p42, LPS 2h, H3K27Ac, ChIP, R3
HPC-7, p42, UT, H3K27Ac, ChIP, R1
HPC-7, p42, UT, H3K27Ac, ChIP, R2
HPC-7, p42, UT, H3K27Ac, ChIP, R3
HPC-7, p30, LPS 2h, INPUT, R1
HPC-7, p30, LPS 2h, INPUT, R2
HPC-7, p30, LPS 2h, INPUT, R3
HPC-7, p30, UT, INPUT, R1
HPC-7, p30, UT, INPUT, R2
HPC-7, p30, UT, INPUT, R3
HPC-7, p42, LPS 2h, INPUT, R1
HPC-7, p42, LPS 2h, INPUT, R2
HPC-7, p42, LPS 2h, INPUT, R3
HPC-7, p42, UT, INPUT, R1
HPC-7, p42, UT, INPUT, R2
HPC-7, p42, UT, INPUT, R3
HPC-7, p30, UT, H3K4me1, ChIP, R1
HPC-7, p30, UT, H3K4me1, ChIP, R2
HPC-7, p42, UT, H3K4me1, ChIP, R1
HPC-7, p42, UT, H3K4me1, ChIP, R2
HPC-7, p30, UT, H3K4me1, INPUT
HPC-7, p42, UT, H3K4me1, INPUT
HPC-7, p30, UT, ER, ChIP
HPC-7, p42, UT, ER, ChIP
HPC-7, p30, UT, INPUT
HPC-7, p42, UT, INPUT
KO-52, Tm, ATF4, ChIP
KO-52, Tm, INPUT
HPC-7, p30, INPUT
HPC-7, p42, INPUT
HPC-7, p30, CEBPa, ChIP
HPC-7, p42, CEBPa, ChIP

Genome browser session
(e.g. UCSC)

Not available

Methodology

Replicates

ChIP-seq experiments for H3K27ac were conducted with three replicates to enable detailed statistical comparisons using DESeq2. For H3K4me, no statistical comparisons were made, so two replicates per sample were used. CEBPA ChIP-seq experiments were done using anti-CEBPA antibody and replicated using anti-ER antibody, to gain confidence in the detection of the over-expressed isoforms. These results were then compared to previously published ChIP-seq data using a CEBPA antibody, thereby enhancing the validity of our findings. ATF4 ChIP-seq in KO-52 was performed with one replicate

Sequencing depth

All samples were paired-end sequenced to a final depth of 20-40M reads/sample.

Antibodies

H3K27ac antibody (Abcam ab4729)
H3K4me1 (Diagenode, C15410194)
ER F-10 (sc-8002X, Santa Cruz Biotechnology)
CEBPA D56F10 (8178, Cell Signaling)
ATF4 B3 (118155, Cell Signaling)

Peak calling parameters

ChIP-seq peaks were identified by MACS295 v. 2.2.5 using default parameters, and using input libraries as control. Peaks identified in each condition were merged with bedtools merge (v. 2.31.1) to generate a common peak set.

Data quality

ChIP-seq reads were pre-processed with TrimGalore v0.6.6 for quality and adapter trimming. Trimmed reads were aligned to mouse genome mm39 using Bowtie293 v. 2.4.4 with '--very-sensitive' flag set and a minimum fragment length (-X flag) of 1000bp, all other parameters set to default. Duplicate reads were identified using Picard MarkDuplicates and excluded from the downstream analysis.

Software

bowtie2 v2.4.4
TrimGalore v0.6.6
MACS2 v2.2.5
bedtools v.2.31.1
Deeptools v.3.31

Flow Cytometry

Plots

Confirm that:

☒ The axis labels state the marker and fluorochrome used (e.g. CD4-FITC).

☒ The axis scales are clearly visible. Include numbers along axes only for bottom left plot of group (a 'group' is an analysis of identical markers).

☒ All plots are contour plots with outliers or pseudocolor plots.

☒ A numerical value for number of cells or percentage (with statistics) is provided.

Methodology

Sample preparation	Cells were first collected, media was washed out and cells were stained with conjugated antibodies in a solution of PBS containing FBS 2% for 20 minutes, then washed and immediately analyzed in the flow cytometer.
Instrument	FACS Canto II (BD Biosciences)
Software	BD FACSDiva v6.1.3<br>FlowJo v10.6.2<br>GraphPad Prism v10.4.1
Cell population abundance	n/a
Gating strategy	Cells were first gated for live cells (FSC/SSC) and gated to exclude doublets by plotting FSC-A and FSC-H. To quantify cell death, cells were stained with Annexin V-APC (BD Pharmingen 550474) and DAPI. Unstained samples were used to draw boundaries, and the same cutoff to quantify Annexin-V positive cells was used for all samples being compared.

☐ Tick this box to confirm that a figure exemplifying the gating strategy is provided in the Supplementary Information.
